# Transcriptome, Spliceosome and Editome Expression Patterns of the Porcine Endometrium in Response to a Single Subclinical Dose of *Salmonella* Enteritidis Lipopolysaccharide

**DOI:** 10.3390/ijms21124217

**Published:** 2020-06-13

**Authors:** Lukasz Paukszto, Anita Mikolajczyk, Jan P. Jastrzebski, Marta Majewska, Kamil Dobrzyn, Marta Kiezun, Nina Smolinska, Tadeusz Kaminski

**Affiliations:** 1 Department of Plant Physiology, Genetics and Biotechnology, Faculty of Biology and Biotechnology, University of Warmia and Mazury in Olsztyn, Oczapowskiego 1A, 10-719 Olsztyn, Poland; lukasz.paukszto@uwm.edu.pl (L.P.); bioinformatyka@gmail.com (J.P.J.); 2 Department of Public Health, Faculty of Health Sciences, Collegium Medicum, University of Warmia and Mazury in Olsztyn, Warszawska 30, 10-082 Olsztyn, Poland; asm@uwm.edu.pl; 3 Bioinformatics Core Facility, Faculty of Biology and Biotechnology, University of Warmia and Mazury in Olsztyn, Oczapowskiego Str 1A/113, 10-719 Olsztyn, Poland; 4 Department of Human Physiology and Pathophysiology, School of Medicine, Collegium Medicum, University of Warmia and Mazury in Olsztyn, Warszawska Str 30, 10-082 Olsztyn, Poland; marta.majewska@uwm.edu.pl; 5 Department of Animal Anatomy and Physiology, Faculty of Biology and Biotechnology, University of Warmia and Mazury in Olsztyn, Oczapowskiego 1A, 10-719 Olsztyn, Poland; kamil.dobrzyn@uwm.edu.pl (K.D.); marta.kiezun@uwm.edu.pl (M.K.); nina.smolinska@uwm.edu.pl (N.S.)

**Keywords:** LPS, RNA-seq, endometrium, alternative splicing, RNA editing, Sus scrofa

## Abstract

Endometrial infections at a young age can lead to fertility issues in adulthood. Bacterial endotoxins, such as lipopolysaccharide (LPS), can participate in long-term molecular changes even at low concentrations. Lipopolysaccharide plays a crucial role in the progression of septic shock, inflammation and auto-immune diseases. The aim of this study was to describe transcriptomic modulations in the porcine endometrium, induced in vivo by a single subclinical dose of LPS from Salmonella Enteritidis. which did not produce clinical symptoms of toxicity. The RNA-seq methodology was applied to reveal 456 differentially expressed regions, including 375 genes, four long noncoding RNAs, and 77 other unclassified transcripts. Two independent methods confirmed 118 alternatively spliced genes that participate i.a., in the formation of the MHC-I complex and the adaptive immune response. Single nucleotide variant-calling algorithms supported the identification of 3730 allele-specific expression variants and 57 canonical A-to-I RNA editing sites. The results demonstrated that the differential expression of genes involved in inflammation, immune response, angiogenesis and endometrial development may be maintained for up to 7 days after exposure to LPS. RNA editing sites and long noncoding RNAs (lncRNAs) play an important role in transcriptional regulatory machinery in the porcine endometrium in response to LPS administration.

## 1. Introduction

The enteric pathogen *Salmonella* Enteritidis is causative agent of salmonellosis [1] and, in rare cases, extraintestinal disorders and septic shock [2]. This facultative intracellular pathogen colonizes specialized compartments in eukaryotic host cells. *Salmonella enterica* is able to survive in host cells by activating cellular stress response systems and highly evolved virulence factors [3]. The cell walls of *Salmonella* and other Gram-negative bacteria contain lipopolysaccharide (LPS) which binds with Toll-like receptors (TLRs) in the eukaryotic cells, promotes the transduction of inflammatory signals and activates the immune system during uterine infections [4]. About 80% of all genital tract infections are induced by *E. coli* [5]. Therefore, most research into LPS has been conducted with the involvement of *E. coli*, whereas *Salmonella* serotypes were far less frequently used [6]. Every year, about 3.4 million worldwide cases of extra-intestinal non-typhoidal *Salmonellae* (NTS) infections are observed [7]. Particularly, the bacteremia is mostly induced by NTS serotypes in sub-Saharan Africa populations [8]. Several known mechanisms participate in crossing the intestinal barrier by the Salmonella species [9]. Recent studies indicate that bacteria are transported by mononuclear cells and they also float as free microorganisms in the bloodstream [10]. Jeon and colleagues postulated that the same core bacterial genera are engaged in common biological networks in uterus and blood [11]. Following this lead, it is assumed that some pathogens may be transmitted to the uterus. Several medical case reports are describing *Salmonella* serotype infections linked with disorders of the reproductive tract and pregnancy complications in humans [12,13]. Moreover, the occurrence of Gram-negative bacteria within the uteroplacental unit increases the possibility of preterm labor or abnormal embryo preimplantation development [14].

The endometrium is the inner layer of the uterus which plays a role in implantation, placentation, and pregnancy maintenance [15]. Microbial uterine infections activate and attract innate immune cells which are indispensable for protecting the female reproductive tract [16]. High concentrations of bacterial endotoxins stimulate the expression and activation of pro-inflammatory cytokines in the endometrial tissues [17]. It has been demonstrated that the LPS-induced dysregulation of prostaglandin secretion is linked with the Toll-like receptor 4 (TLR4) signaling pathway, which explains the prolonged luteal phase in the bovine uterine disorders [18]. Dynamic changes in the uterine microenvironment during LPS administration were observed in many species [4,17,18,19], including pigs [20,21].

It has been found that LPS at a low dose (5 μg/kg b.w) enhances fluctuations in gene and protein expressions in the porcine adrenal glands, gallbladder, brain and nervous system [22,23,24,25]. During pregnancy, the exposure to LPS at a high dose induces the intra-uterine fetal death [26]. However, even low doses of LPS can effectively induce inflammatory mediators such as PGE2_,_ TNF-α, IL-1β, IL-6 and INFs [27,28]. There is evidence to suggest that low doses of LPS increase the expression of proinflammatory molecules without inducing septic shock, and this state is often called “endotoxin tolerance” [29]. After infection, most LPS molecules are cleared from the host, but ~20% of LPS may still circulate in the bloodstream for a long time and interact with the innate immune system [30]. Most studies on pigs focused on LPS injected at moderate or high doses and no longer than 12 h after administration, precluding the possibility of discovering its long lasting effects on animal or human physiology [31].

The effect of bloodborne *Salmonella* LPS on the transcriptome of the porcine endometrium has not been studied to date. Research into genome-wide expression profiling has analyzed differentially expressed genes (DEGs), alternative splicing events, expression polymorphisms and expression targets to identify the links between various physiological or pathophysiological statuses and genetic backgrounds. Multilayered and deep genetic investigations of the host–pathogen interaction are a prelude and necessary step for proteomic and metabolomic approaches, that will bring us closer to the clinical implementations. The basic gene expression profiles and their changes are only a small part of the genetic overview of the experiments. Developing high-throughput sequencing methods has proven to be growing role of junk RNA and alternatively spliced transcripts.

The aim of this experiment was to describe in vivo genetic modifications in the endometrium of pigs administered a low single dose of LPS from *Salmonella* Enteritidis. No noticeable clinical symptoms of intoxication after 7 days of the LPS injection can hypothetically take place during the asymptomatic carrier state of *Salmonella* spp. A long-term contact with the pathogen, in particular with a bloody supply tissue/organ such as the uterus, may also lead to the host adaptive immune response. This study focused on a long-term impact of LPS on the porcine endometrial transcriptome induced by contact with the pathogen.

## 2. Results

### 2.1. Mapping and Clustering of RNA-Seq Libraries

**Figure 1:**
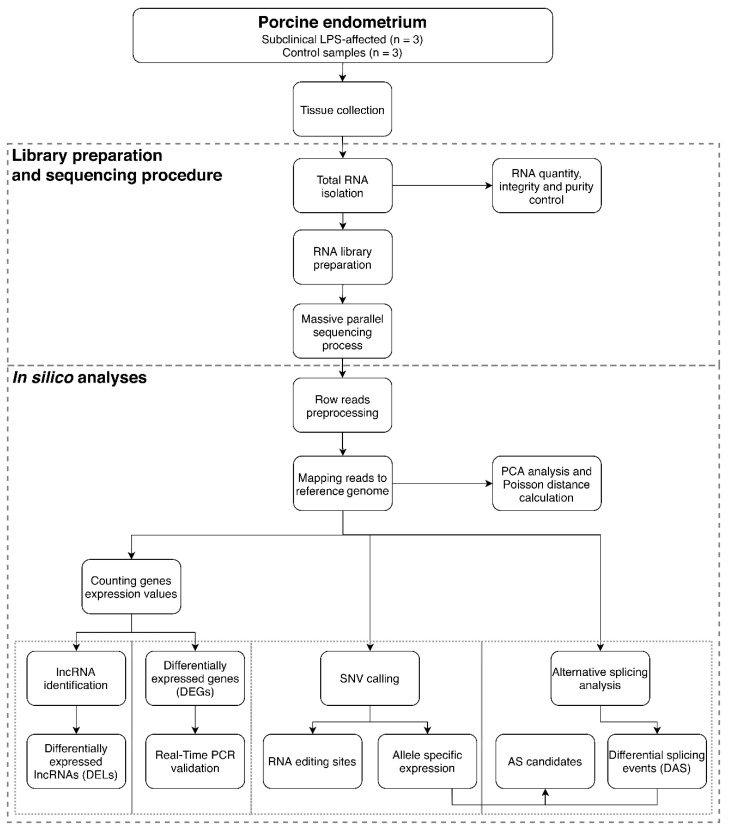
Flowchart of the RNA-seq wet-lab and in silico analyses. The main analytic pipeline contains: preprocessing quality control, mapping reads, biological replicates validation, differential genes and long noncoding RNAs (lncRNAs) expression analysis, alternative splicing events identification, allele-specific expression, RNA editing sites and real-time PCR validation. LPS—lipopolysaccharide; SNV—single nucleotide variant; AS—alternative splicing; PCA—principal component analysis.

Created RNA-seq libraries were sequenced in three biological replications for LPS-treated and control groups (Figure S1). Transcriptome-wide analysis of the effects of LPS administration on the endometrium was described on pipeline (Figure 1).

**Figure 2:**
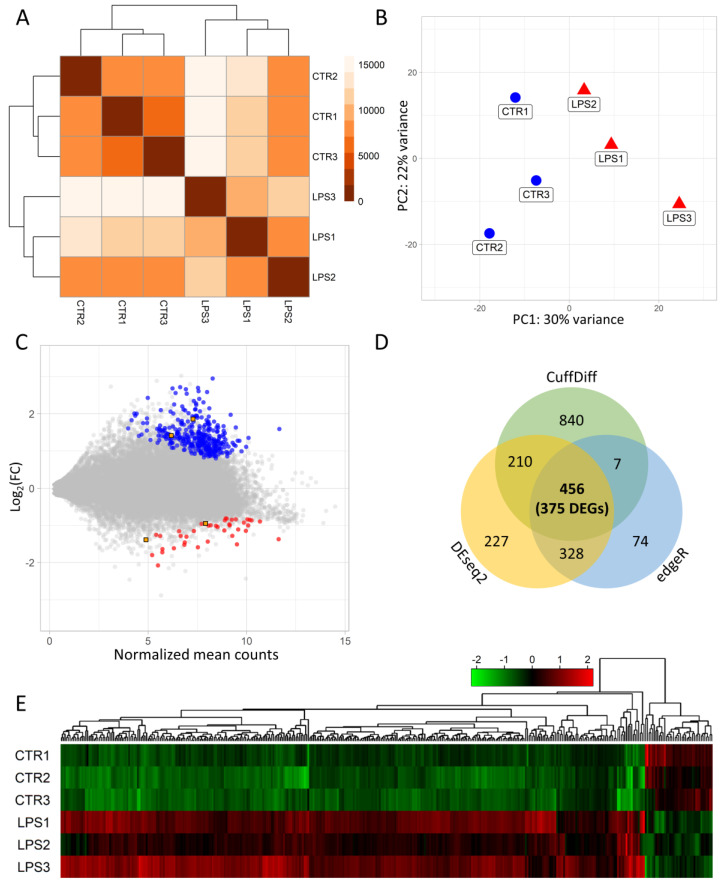
Transcriptome-wide RNA-seq analysis and expression profiles of the differentially expressed genes (DEGs) and lncRNAs (DELs) after LPS administration in endometrium. (**A**) The Poisson distance matrices of the transcriptomic profiles in the porcine endometrium treated by LPS. (**B**) Principal component analysis (PCA) of 6 RNA-seq libraries isolated from the endometrium (3× treated by low dose of LPS—red triangles; and 3× controls—blue circles); *X* axis describes the principle component 1 and *Y* axis depicts principle component 2 results; (**C**) MA plot presents the logarithmic scale of the fold changes in the *Y* axis and the normalized expression count values in the *X* axis. Blue dots represent upregulated DEGs, red dots describe downregulated DEGs and orange rectangles depict significant DELs. (**D**) Venn diagram of differentially expressed transcriptionally active regions (DE-TARs). Numbers in circles represent DE-TARs classified by DESeq2, Cufflinks and edgeR. Middle numbers include DE-TARs and DEGs intersected by those methods. (**E**) Heatmap of DEGs based on the the Z scores scale. Columns in heatmap represent DEGs; rows indicate individual RNA-seq libraries.

HiSeq 4000 (Illumina) sequencer generated 282,664,081 raw paired-end reads (Table 1). Preprocessing quality control procedure survived 260,974,918 clean reads, and of them, 216,501,753 were uniquely mapped to the reference porcine genome (Sscrofa11.1.91). The mean percentage distribution of aligned reads to the genome was as follows: 36% to CDS regions, 24% to UTRs regions, 5% to introns and 35% to intergenic locations (Table S1). We identified 21,054 transcriptionally active regions (TARs). in the porcine endometrium, that were expressed in at least half of the examined samples. The calculation of te Poisson distance (Figure 2A) and principal component analysis (PCA) (Figure 2B) revealed that the expression pattern of the samples clustered according to biological replicates within the LPS-treated (n = 3) and non-treated (n = 3) groups.

### 2.2. Differentially Expressed Genes (DEGs) and Functional Annotations

**Figure 3:**
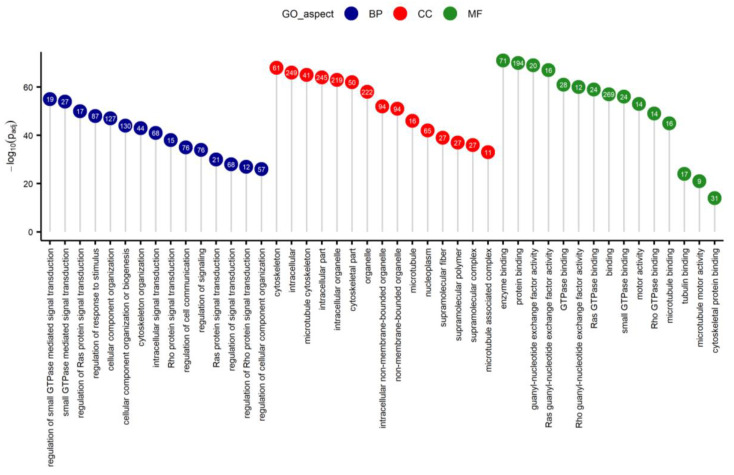
Lollipop chart of the top 15 Gene Ontology (GO) terms for each GO aspect (BP—biological process; CC—cellular components; MF—molecular function), scattered according to −log10 adjusted *p*-value. Numbers in circles refer to the number of DEGs annotated to the specific GO term.

Three methods, Cufflinks, DESeq2, and edgeR detected 1513; 1221 and 865 differentially expressed TARs (adjusted *p*-value < 0.05), respectively, and 456 TARs were confirmed by all three methods (Figure 2D). The expression profiles of all transcripts were described by normalized counts values, logarithmic fold change (log2FC) and adjusted *p*-value in MA plot (Figure 2C). Within the differentially expressed TARs, 375 DEGs showed statistically significant changes in the expression between the LPS-treated and the control samples (Table S2). The DEGs were visualized in a heatmap (Figure 2E). The 336 DEGs demonstrated overexpression and 39 were downregulated in the samples from the LPS group. The top five upregulated protein-coding genes were: EGFR (logFC = 2.95), SIK1 (logFC = 2.72), CEP250 (logFC = 2.70), ARHGAP33 (logFC = 2.68), CDC42BPG (logFC = 2.66). Otherwise BTNL9 (logFC = −1.61), SIX1 (logFC = −1.64), TMEFF2 (logFC = −1.72), S100A9 (logFC = −1.80), and CCL23 (logFC = −2.08) genes, which showed the highest downregulation in LPS-treated samples (Table S2). Among all DEGs qualified to enrichment functional analysis, 313 were assigned to 24 biological processes (BP), 17 molecular functions (MF) and 26 cellular components (CC) gene ontology annotation terms (Figure 3, Table S3).

**Figure 4:**
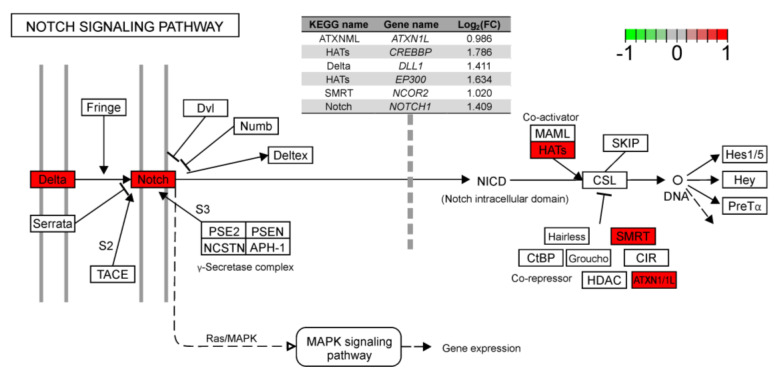
Enrichment Kyoto Encyclopaedia of Genes and Genomes (KEGG) analysis of DEGs engaged in the Notch signaling pathway.

Within the BP category, the most interesting DEGs were enriched to several subcategories: ‘regulation of Ras protein signal transduction’, ‘regulation of response to stimulus’, ‘Rho protein signal transduction’ and ‘neurogenesis’. The MF aspect encompassed genes annotated to significant ontology terms, for example: ‘Ras/Rho GTPase binding’, ‘enzyme binding’ and ‘microtubule motor activity’. Additionally, proteins coded by DEGs were cellular components of e.g., cytoskeleton, microtubule and collagen trimers. Metabolic pathways analysis revealed two significant Kyoto Encyclopaedia of Genes and Genomes (KEGG) items (‘Thyroid hormone signaling pathway’ and ‘Notch signaling pathway’), that changed due to LPS injection. All six genes (ATXN1L, CREBBP, DLL1, P300, NCOR2, NOTCH1) assigned to the Notch signaling pathway were upregulated (Figure 4).

Among the genes engaged in the TLR4 signaling pathway, only TRAF3 indicated significant upregulation in the samples from the experimental group.

The results revealed the dysregulation of the six crucial biomarkers involved in endometritis: SLC7A5, COL1A1, AXIN2 (upregulated); CFD, MGP, CCDC3 (downregulated). The other important DEGs were involved in the synthesis of fatty acids (FASN, logFC = 1.66), signal transduction pathways (CREBBP, logFC = 1.79; p300, logFC = 1.63), angiogenesis (EGFR, logFC = 2.95; VEGFA, logFC = 1.26) and defensins production (PLA2G4B, logFC = 1.12; ALOX15, logFC = 1.71).

### 2.3. Long noncoding RNA (lncRNA) Identification and Cis/Trans-Connection with Protein-Coding Genes

In the first filtration step, after removing protein-coding genes, sequences shorter than 200 bp and one-exon transcripts from the annotated dataset, we detected 1802 potential lncRNA candidates expressed in the porcine endometrium. The obtained sequences were scanned for coding potential by four methods (CPAT, CPC2, FEELnc, Pfam) and 1072 unannotated transcripts were indicated as lncRNA biotype. Neither of the identified transcripts matched to small RNA models in the Rfam database. Identified novel lncRNA transcripts and 113 known lncRNAs were located in 773 non-coding regions. To examine the transcription regulatory machinery during LPS administration in the porcine endometrium, we retrieved two (MSTRG.3558, MSTRG.19846) upregulated and two (MSTRG.9270, MSTRG.15739) downregulated lncRNA loci (Table 2).

Only upregulated differentially expressed lncRNAs (DELs) showed trans-interaction with 30 target genes, due to the similarity of expression profiles (absolute value of the Pearson correlation coefficient >0.9) (Table S4). Trans-interactions revealed 26 and 17 potentially regulatory target genes for MSTRG.3558 and MSTRG.19846, respectively. Only one gene (C1QA) was positively regulated by MSTRG.9270 lncRNA. The 155 genes were located in the vicinity (<2000 bp) of 152 lncRNA (Table S5). Neither of the cis-acting lncRNA were identified in DELs dataset.

### 2.4. Differentially Alternative Splicing (AS) Events

**Figure 5:**
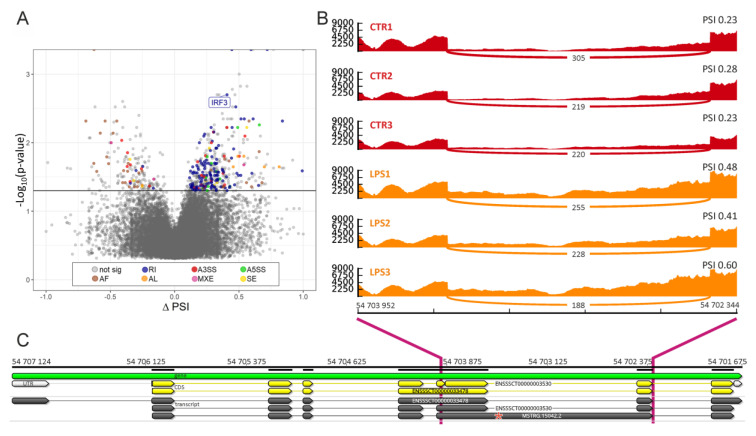
The distribution of differentially alternative splicing (DAS) events. (**A**) Volcano plot shows the difference in the percent splicing inclusion (ΔPSI) values in each alternative splicing (AS) event (*X* axis) and the *Y* axis depicts the logarithmic adjusted *p*-values. The horizontal line describes adjusted p-values cut-off (0.05). Colors of the dots indicate specific types of DAS: skipping exon (SE—yellow), alternative 5′ splice site (A5SS—green), alternative 3′ splice site (A3SS—red), mutually exclusive exons (MXE—pink), retained intron (RI—blue), alternative first (AF—brown), last exon (AL—orange), and not a significant event (grey). (**B**) Quantitative visualization (Sashimi plot) of the intron 5 (I5) retention within the IRF3 gene. Each track visualizes the splicing event (RI of I5) within the biological replicates for the control (red) and LPS-treated (orange) samples. Count values on curved lines describe the coverage within the splice junction. The left scale presents the coverage depth of IRF3 in the range of the AS region. PSI values are presented on the right side for each track. (**C**) Genomic coordinates of the DAS event within IRF3 located on chromosome 6. Yellow tracks show 2 known ENSEMBL coding regions (CDS) of the IRF3 gene. Grey tracks show 2 known and 1 novel transcripts of IRF3 identified in our study. Red star shows the position of the stop codon in the alternative spliced IRF3 mRNA (MSTRG.15042.2) after intron retention.

Two approaches (rMATS and SUPPA) permitted to detect 45,023 and 45,217 splicing events. Both methods also supported the identification of 4605 (rMATS) and 633 (SUPPA) differentially alternative splicing (DAS) events, as a result of the comparison of treated and control samples. However, the consensus of both methods confirmed 192 and 214 DAS events, according to the intersection of genomic localization, respectively, for rMATs and SUPPA (Table S6). All DAS events were assigned to 118 protein-coding genes. Among the detected DAS events, eight were classified as skipping exons (SE), 19 as alternative 5′ splice sites (A5SS), 25 as alternative 3′ splice sites (A3SS), nine as mutually exclusive exons (MXE), and 131 as retained introns (RI) according to rMATS classification. SUPPA grouped 214 DAS events to seven types: eight (SE), 11 (A5SS), 24 (A3SS), three (MXE), 129 (RI), 33 (alternative first—AF) and six (last exon—AL) (Figure 5A, Table S6).

Most of the DAS events identified using both methods shared the same type of alternative splicing (AS). Discovered DAS genes were annotated to 11 Gene Ontology (GO) terms. The findings indicated that some genes (ENSSSCG00000001398, ENSSSCG00000001341, CD1D, FCGR1A, ADA) were enriched to ‘antigen processing and presentation via MHC class Ib’ and ‘positive regulation of adaptive immune response’ (Table S7). Most of the important genes with identified DAS events, i.a., SIGIRR (ΔPSI = 0.291), HOXB7 (ΔPSI = 0.242), LIN7B (ΔPSI = 0.267) and IRF3 (ΔPSI = 0.408) (Figure 5B,C), revealed higher percentage splicing inclusion in LPS-treated samples. Otherwise, a higher percentage of exclusion was evaluated for CPT1B (ΔPSI = −0.344), TGIF1 (ΔPSI = −0.258) and SURF4 (ΔPSI = −0.534). Furthermore, the three DEGs (CAPN15, ZSWIM8, AKAP17A) showed a higher percentage of the transcript with intron retention (RI) in the LPS-treated samples. Moreover, the CALU, CDK9, CIRBP, GPAA1, NAGK, NUDT14, STX18 and TOM harboured multiple types of DAS events within the junction sites, preferably noticed in the transcripts exposed to LPS.

### 2.5. Allele-Specific Expression (ASE)

**Figure 6:**
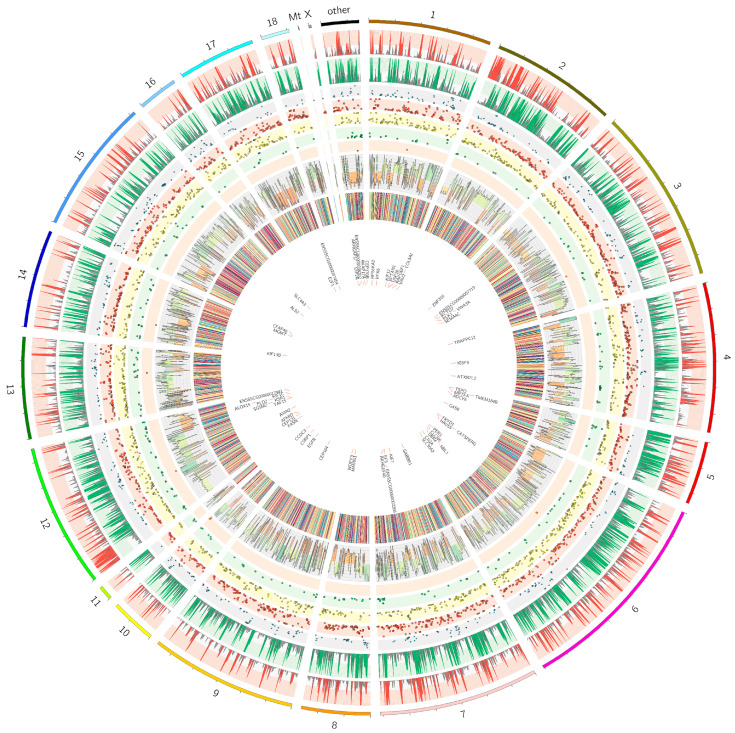
Visualization of the allele-specific expression (ASE) sites exposed to the low dose of LPS. The external circle represents the porcine chromosomes and scaffolds, where each chromosome length describes the number of ASEs. Two upper tracks depict the coverage of the reference (red histogram) and alternative variants (green histogram) in all the RNA-seq libraries. The next five middle scatter plots show the localization of ASE on intergenic (blue triangles), UTR (red squares), CDS (yellow dots), intron (green squares on green background) and ncRNA region (orange squares on orange background); vertical axis on each scatter plot shows the normalized FDR. The histogram visualizes a difference in the alternative allele fraction (ΔAAF) between the LPS-treated samples and the controls. In this track, green indicates a higher AAF in the controls and orange represents a higher AAF in the LPS samples. The tenth track (12-color barcode plot) presents an abundance of 12 types of nucleotide substitutions. The most inner track depicts the names of 55 DEGs with significant ΔAAF in ASFE sites.

Based on six RNA-seq libraries, 153,269 single nucleotide variants (SNVs) were called for the porcine endometrium transcriptome. Using standard GATK filters, 135,293 substitutions/sites survived the first step of the quality pipeline. Then, we obtained 86,972 polymorphic sites after removing the SNVs located in the range of bidirectional genes, simple sequence repeats, paralog regions and in the vicinity of splice junction sites. Finally, 53,472 SNVs possessed an alternative allele fraction in at least three RNA-seq libraries. After the filtering procedure, 3730 ASE candidates displayed a significant imbalance in allele expression (alternate allele frequency (AAF) > 0.1; false discovery rate (FDR) < 0001) between the LPS-treated and the control samples (Figure 6; Table S8). According to the variant effect predictor (VEP) annotation, 865 ASE sites were depicted in downstream regions, 244 in upstream regions, 1065 on 3′UTR, 67 on 5′UTR, 283 within the intronic regions and 1074 on CDS sequences. Additionally, heterozygous ASEs were appraised as 2296 “heteroref”, 1210 “heteroalt” and 224 “true” heterozygotes. ASE variants were identified in the range of 18 DAS genes and 55 DEGs (Figure 6).

### 2.6. RNA Editing Prediction

The 1422 polymorphic sites (with AAF < 0.7 in all samples and no annotation in dbSNP) were assigned to RNA canonical substitutions (A to I and C to T). The fifty-three substitution sites were localized within Sus scrofa PRE-1 SINE regions, and these SNVs were qualified as RNA editing candidates. Additionally, twenty-two of them were confirmed in the pig editome DREP database. Adenosine to inosine (‘A to I’) RNA editing candidates were classified as 21 UTR, 14 downstream, 17 intronic and five upstream variants (Table 3). Finally, six of the RNA editing sites (located in the vicinity of SPPL2A, RSAD1, GFM1, ENSSSCG00000031589, TMEM161A, GNG3) also indicated significant (FDR < 0.05) imbalance in allele fraction.

### 2.7. Real-Time PCR Validation

**Figure 7:**
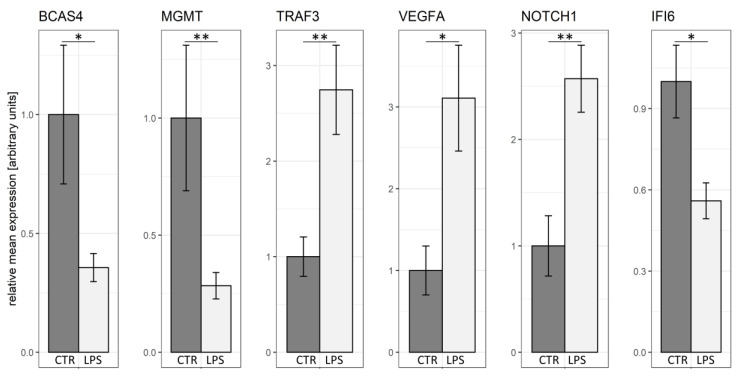
Real-time PCR validation of the RNA-seq results for the DEGs in the endometrial cells. Validation was performed for BCAS4, MGMT, TRAF3, VEGFA, NOTCH1 and IFI6 genes with reference genes (ACTB and PPIA). LPS—samples from the LPS-treated groups; CTR—samples from the control groups; * *p*-value < 0.05; ** *p*-value < 0.01.

To validate the obtained RNA-seq results, six DEGs (BCAS4, MGMT, TRAF3, VEGFA, NOTCH1, IFI6) were selected for qRT-PCR. The qRT-PCR expression patterns of these DEGs agreed with the RNA-Seq results. qRT-PCR confirmed the veracity and accuracy of the high-throughput methods used in the present study (Figure 7).

## 3. Discussion

This study made the first ever attempt to describe transcriptomic changes in the porcine endometrium resulting from the exposure to a subclinical dose of *Salmonella enterica* LPS. A total of 375 protein-coding genes and four differentially expressed lncRNA loci were identified. The functional annotation of the analyzed genes demonstrated that DEGs (336 up-regulated and 39 down-regulated) were involved in, among others, the Notch signaling pathway, the Rho protein signal transduction and cytoskeleton formation. An analysis of alternative splicing events revealed 118 protein-coding genes with significant changes in exon and intron inclusion levels. The SNV calling approach revealed 3730 SNVs with differences in alternative allele fraction and 57 A-to-I canonical RNA editing candidates, which could play a regulatory role in response to a subclinical dose of LPS.

Lipopolysaccharide is produced by Gram-negative bacteria, and it plays a crucial role in the activation of pro-inflammatory cytokines which dysregulate the uterine microenvironment [32,33]. Neonatal exposure to LPS may have consequence in immune responses later in life [34]. Therefore, we may assume that LPS administration at a young age may be involved with uterine welfare in adulthood. This study proposes a model of a single low dose of *S. enterica* LPS injection to the uterus, without phenotypic symptoms of illness. The molecular pathways associated with the endometrial immune response to the presence of the pathogen in the bloodstream are still unknown. This study set out to investigate whether, and if so, which molecular mechanisms have been induced to suppress transcriptional responses leading to inflammation, after 7 days of infection. There is evidence to suggest that some pro-inflammatory cytokines may be engaged in normal uterine functioning during the menstrual/estrous cycle and early pregnancy [35,36]. However, gilts reach sexual maturity at around five/six months of age. Therefore, the confounding effect of the estrous cycle was disregarded, and only the symptoms triggered by LPS were investigated.

Innate immunity and mucosal defense systems are activated in the presence of bacteria in the female genital tract. Lipopolysaccharide is the main component of bacterial cell walls which induces the expression of TLR4 receptors, and consequently, activates the NF-ĸB transcription factor and stimulates the production of inflammatory cytokines [37]. Toll-like receptors mediate the production of cytokines and growth factors, as well as the proliferation of endometrial cells in response to LPS [38]. In the present study, the expression of most genes whose products are engaged in the TLR4 pathway was not significantly altered in the endometrium of pigs exposed to LPS. Only *TRAF3* (logFC = 1.81) was upregulated in LPS-treated animals. There is evidence to indicate that TRAF3 is an important signaling protein which participates in the inhibition of inflammatory cytokine production. TRAF3 has to be degraded to activate the MAPK signaling pathways and effector functions of the immune system [39]. Previous studies of bovine endometrial cells revealed that IRF3 depletion may inhibit the RIG-I signaling pathway [40]. The present study demonstrated that even the overexpression of *TRAF3* did not affect the expression profile of *IRF3* in the examined samples. However, alternative splicing analysis revealed higher inclusion levels of *IRF3* intron 5 (I5) in LPS-treated samples, which could be attributed to the formation of more inactive IRF3 isoforms during translation. This study also revealed the presence of intron-retaining *SIGIRR* transcripts in the treated samples. Shorter mRNA products suppress the activation of SIGIRR protein, which could be linked with an immune tolerance to some microbial components after the silencing of the TLR signaling pathway [41]. The deactivation of JNK and p38 family members (members of MAPK kinase signaling cascades) may be a part of negative feedback involving *DUSP8* [42]. The expression of *DUSP8* was altered (logFC = 1.44), which may mute MAPK and Toll-like signaling pathways.

The gene ontology enrichment analysis revealed the importance of the Ras/Rho protein signal transduction and Ras/Rho GTPase activation, after LPS administration in the porcine endometrium. Ras proteins activate all three types of MAPK in vascular smooth muscle cells, which generally increases cytoplasmic phospholipase A2 (*cPLA2*) expression [43] and/or cytokine production [44]. The activation of Rho is associated with the LPS/NF-kB signaling pathway. Virulence factors, including LPS, affect and interact with the actin cytoskeleton and RhoGTPases. The Rho signaling pathways also control trophoblast invasion in the endometrium [45]. This study also revealed the overexpression of *RhoF* (logFC = 1.32) and two *CDC42* downstream effectors, *CDC42BPB* (logFC = 1.27) and *CDC42BPG* (logFC = 2.66), which cooperate in cytoskeletal reorganization. *CDC42* and *EGFR* have been used as potential targets for angiogenesis and cancer therapies [46]. These findings suggest that the instability of the genes involved in Ras/Rho signaling pathways, in the endometrium of pre-pubertal gilts exposed to LPS, could lead to reproductive disorders.

Angiogenesis plays an important role in endometrial receptivity [47]. In this study, LPS treatment influenced several genes, including *EGFR* (logFC = 2.95), *VEGFA* (logFC = 1.26) and *NOTCH1* (logFC = 1.41), whose products are involved in angiogenesis control. The activation of EGFR induces *VEGF* overexpression in proangiogenic cells during the formation of new vessels [48]. Vascular endothelial growth factor (*VEGF*) signaling is strongly linked with *NOTCH1* expression [49]. Endometrial tip cell migration and stalk cell proliferation are dependent on molecular cross-talk between VEGF and NOTCH1 [50]. A deficiency of both factors can induce arterial defects [51]. The involvement of the NOTCH signaling pathway in the overexpression of the *NOTCH1* receptor and its ligand *DLL1* plays a fundamental role in the development of endometrial arteries and maximizes reproductive success in mice [52]. The LPS injection also upregulated the *ALOX15* gene (logFC = 1.71) whose product controls cytokine generation [53], induces the proliferation of tumor cells and the dysregulation of angiogenesis. The adverse effects of *ALOX15* can be mitigated by the overexpression of *cPLA2*. Cytoplasmic PLA2 releases arachidonic acid from the cell membrane which stimulates vein growth [54]. PLA2 also stimulates defensin production and plays a crucial role in the inhibition of bacterial enzymes [55]. This study revealed that *ALOX15* and *PLA2G4B* (logFC = 1.12) were upregulated in response to LPS, which suggests that LPS participates in both the disruptive and repair mechanisms of angiogenesis. These observations suggest that subclinical doses of LPS exert a more or less direct effect on angiogenesis in the porcine endometrium.

In many cases, a bacterial infection of the uterus can lead to clinical or subclinical endometritis [56,57]. This study confirmed differences in the expression profiles of six biomarkers in LPS-induced bovine endometritis: *SLC7A5*, *COL1A1*, *AXIN2* (upregulated) and *CFD*, *MGP*, *CCDC3* (downregulated) [57]. *COL1A1* overexpression was also observed in the eutopic endometrium of infertile women with endometritis [58]. The presented experiment also revealed differences in the alternative splicing of *TGIF1* induced by LPS administration. This gene was significantly dysregulated in cows with clinical and subclinical endometritis [57]. The discussed genes could play a key role in the molecular mechanisms underlying endometritis. It is worth noting that several transcripts (e.g., *CD1D* and *ENSSSCG00000001398* encode MHC class I antigen 7, *FCGR1A* and *ADA*) enriched in the MHC class I protein complex, and the ‘positive regulation of adaptive immune response’ were significantly modified by alternative splicing. The overexpression of yjr MHC-I complex is linked with low fertility and endometritis [59]. Long-term endometritis may lead to impaired implantation [60] or cancerogenesis [61].

Research into human decidual cells has demonstrated that LPS influences the expression of genes engaged in lipid metabolism, including cholesterol and steroid biosynthesis [62]. Proprotein convertase subtilisin/kexin type 9 (product of *PCSK9*), the key regulator of serum cholesterol levels, inhibits LPS removal from the bloodstream [63]. The overexpression of *PCSK9* reduces LPS clearance and may exacerbate tissue inflammation during sepsis [64]. Interestingly, a subclinical dose of LPS induced *PCSK9* upregulation (logFC = 1.60), which could have contributed to endometritis. A low LPS dose stimulated the fatty acid synthase gene (*FASN*; logFC = 1.66) which is linked with cholesterol synthesis and lipid raft formation [65]. The similarities in the *FASN* expression profiles in the adrenal glands of the same pigs [22] suggest that *FASN* is one of the fifteen genes that could act as a molecular signature of exposure to low LPS doses in pigs.

The administration of LPS also altered the expression of genes whose products are present in many hormonal signal transduction pathways. This study revealed the overexpression of genes encoding two coactivators, E1A Binding Protein P300 (*p300*; logFC = 1.63) and CREB Binding Protein (*CREBBP*; logFC = 1.79), as well as nuclear receptor corepressor 2 (*NCoR2*; logFC = 1.02). These factors are responsible for recruiting estrogen, progesterone and thyroid hormone receptors, and they play a critical role in embryonic development [66]. Subclinical doses of LPS can lead to dysfunctions of the signaling pathways and receptors.

The analysis of the interactions between *trans*– and *cis*-acting lncRNAs and mRNAs revealed three lncRNAs responsible for the positive regulation of protein-coding genes at independent loci and 212 lncRNAs located in the vicinity of target genes. A comprehensive analysis of lncRNA profiles revealed uncovered *MSTRG.19846* and *MSTRG.3558* lncRNAs that positively regulated DEGs (*CREBBP* and *p300*). *MSTRG.3558* was also a *trans*-acting element of the *TRIO* gene which influences the Ras protein that participates in the signal transduction and cell growth [67]. The downregulation of the third lncRNA, *MSTRG.9270*, may inhibit the expression of *C1QA*, whose product is a component of the immune complement system [68]. Acting as *cis*-elements, lncRNAs may modulate the expression of closely related genes such as *HMGB2* and *SEC23B*, which are implicated in nucleic acid-mediated TLR responses [69] and endometrial abnormalities [70], respectively. Long noncoding RNAs exhibit precise tissue-restricted expression, but in this study, we identified 112 expressed lncRNAs that are commonly encountered in both the endometrial and adrenal cortex tissues of pigs [22]. This study also confirmed the expression of 26 lncRNAs that had been identified in the porcine endometrium by Wang et al. [71]. This is the second study to demonstrate that subclinical doses of LPS induce changes in lncRNAs in porcine tissues, but it is the first ever study to examine the noncoding transcriptome of the endometrium under exposure to LPS. Our findings suggest that lncRNAs are important regulators of endometrial response to subclinical LPS doses, but DELs appear to play a key role in porcine adrenal glands [22].

There is still a broad range of unknown SNVs with unresolved functions and without specific annotation in the porcine genome. For this reason, there is a need to broaden and complement these ASE- and RNA editing catalogs by investigating every single SNV/SNP expressed in specific tissues essential e.g., for reproductive health and disorders. An analysis of alternate allele frequency (AAF) revealed that 3730 SNVs (FDR < 0.001) were involved in the endometrial response to a subclinical LPS dose. In the group of the identified ASE, 288 missense substitutions could lead to changes in protein sequence, folding and structure. Two nonsynonymous changes were observed in *HMGB2* and *PAF1* genes that are directly involved in the cellular response to LPS. Higher AAF of rs80832658 and rs326056210 in the LPS-treated samples could modulate the activation of both genes. There is evidence to indicate that the PAF protein family offers protection against LPS-induced endotoxemia [72]. A novel missense variant in *AXIN2* and a variation (rs699379642) in *PMS2*, the biomarkers of uterine abnormality, have been identified in this study. The expression of *PMS2* mutation has been found to increase the risk of endometrial cancer [73] and *AXIN2* polymorphisms may activate the Wnt pathway associated with reproductive tract carcinomas [74].

In primates, RNA editing events are located in the Alu region [75,76], but the pig genome is devoid of these repetitive regions [77]. After the multistep filtering procedure, we focused on 57 A-to-I editing sites located in PRE-1 SINE regions. The canonical A-to-I editing sites represent more than 90% of all mismatches in the repetitive regions such as the SINE region [77]. Our previous research [22] and the present study revealed that A-to-I editing sites occurred more frequently in the proximity of the 3′UTR of the *SPPL2A* gene in samples from the LPS-treated group. These findings imply that *SPPL2A*, which participates in the immune response, may be specially activated through RNA editing mechanisms, when Gram-negative bacteria infect different types of tissues. Comparing our results with the atlas of the RNA editome in *Sus scrofa* skeletal muscle [78], we confirmed 22 of 57 RNA editing sites expressed in the endometrial tissues. Significant AAF changes in 3′UTR *TMEM161A* and upstream from *GFM1* could be linked with the potential mechanism of implantation failure [79] and intrauterine growth retardation [80], respectively. SNV-calling methods are valuable tools which expand our understanding of post-transcriptional modifications in the porcine genome.

It is worth mentioning that LPS may induce different biological effects dependently on the concentration of the endotoxin used. The limitation of our experimental model is the application of only one single low dose of LPS, and only one time point for the collection of tissue samples. The described above effects of LPS treatment may be due to the action of LPS molecules still circulating in the blood one week after administration, but it is also plausible that the LPS influence on uterine transcriptome partly disappeared after this time.

Deeper sequencing supports detailed analyses of the molecular mechanisms underlying essential physiological and pathological processes. Next-generation computational algorithms facilitate big biological data analyses. This study demonstrated that a subclinical dose of *S. enterica* LPS may induce significant transcriptomic changes that are involved in endometritis, immune response, angiogenesis and endometrial development. The gene ontology analysis of differentially spliced genes revealed the group of transcripts that play an important role in the adaptive immune response and antigen presentation via MHC-I. Our findings also indicate that tissue-specific transcriptomic regulatory mechanisms are activated in response to LPS administration. In the future, the impact of low-LPS doses on the endometrium should be explored with the use of single-cell RNA-seq methods to elucidate the expression patterns in different types of cells with established toxin tolerance.

## 4. Materials and Methods

### 4.1. Ethics Statement and Collection of Samples

The experiment was approved by the Local Ethical Committee for Animal Experimentation in Olsztyn, Poland, which is part of the National Ethics Commission for Animal Experimentation of the Polish Ministry of Science and Higher Education in Warsaw, Poland (Decision No. 73/2015 of 29 September 2015). The breeding and collection procedures were conducted in observance of all institutional guidelines applicable in the Republic of Poland according to the provisions of the Act of 15 January 2015 on Animal Welfare in Science and Education (Dz.U.2015.0.266). Six clinically healthy pigs (Pietrain × Duroc female) with a body weight of 16–18 kg were randomly divided into two groups: LPS (experimental) and control group. The immature pigs were used to avoid the effect of hormones associated with the estrus cycle on the uterine transcriptome. The control animals were injected with saline solution, while the pigs of the experimental group received LPS from *Salmonella* enterica serotype Enteritidis (catalogue No. L7770, Sigma Aldrich, Germany). Animals were intravenously injected (into the marginal ear vein) with LPS at a dose of 5 μg/kg b.w. (in 10 mL saline solution) for the treated group and with a 10 mL saline solution for control group. For seven days after the intravenous injection of LPS, the pigs did not exhibit any symptoms of illness. After seven days of LPS administration, the animals were anesthetized with propofol (Scanofol, NORBROOK, Northern Ireland) and euthanized with pentobarbital (Morbital, Biowet Puławy Sp. z o.o, Poland) [22]. The tissue sections of endometrium were collected immediately after euthanasia, washed in 0.9% NaCl, frozen in liquid nitrogen and stored at −80 °C until total RNA extraction. All information about the animal breeding, procedures of LPS injection and the health status of the animals belonging to the LPS and control groups were described previously [25,81,82].

### 4.2. Library Preparation and RNA-Seq Sequencing

Global expression analysis was performed for the porcine endometrium of both the control (n = 3) and experimental (n = 3) groups. Briefly, the uteri of piglets were collected seven days after subclinical LPS administration. RNA extractions were described previously by Paukszto and coauthors [22]. To identify the expression profiles of the treated pigs, cDNA libraries were performed by TruSeq RNA Sample Prep Kit v2 with the unique pairs of adapters for each sample and RNA-seq was performed on the HiSeq 4000 Illumina platform (Illumina, San Diego, CA, USA). The raw data were submitted to the European Nucleotide Archive (ENA) under accession No. PRJEB37538.

### 4.3. Transcriptome Expression Profiling

Generated 2 × 100 bp paired-end reads were evaluated with FASTQC software version 0.11.7 [83]. Trimmomatic software v. 0.38 [84] was used for Illumina adaptors and poly(A) stretches removal (PHRED cutoff score ≤ 20; calculated on both ends of reads and with 10 bp frameshift). All reads were trimmed to equal length (90 bp). After checking the quality, the paired-end reads were mapped to the pig reference genome with ENSEMBL/GENCODE annotation (Sus_scrofa.Sscrofa11.1.91) using the STAR mapper [85]. StringTie v. 1.3.3 [86] pipeline was used to annotate and estimate the expression of porcine genes and uncovered regulatory transcriptionally active regions (TARs). The integrity within the control and experimental RNA-seq libraries was verified by PoiClaClu and pheatmap Bioconductor packages of R software v.3.6.2 [87]. To identify the differentially expressed genes (DEGs), we applied the following statistical methods: Cufflinks [88], DESeq2 [89], edgeR [90]. The final consensus results constituted significant DEGs with known protein-coding annotation, confirmed by both statistical tests (adjusted *p*-value < 0.05). Enrichment ontology and pathway analysis were performed with the use of gProfileR [91] based on gene ontology (GO) [92], and the Kyoto Encyclopaedia of Genes and Genomes (KEGG) [93] databases. DEGs were illustrated in an MA, a Volcano and a heatmap plots with gplots Bioconductor package and a custom script in R.

### 4.4. Identification and Expression Profiling of Long Noncoding RNA (lncRNAs)

The multi-stage workflow was implemented for lncRNA identification in the porcine endometrium. Firstly, protein-coding and low-expressed transcripts were excluded from the downstream analysis. Secondly, short (length < 200 bp) and one-exonic transcripts were also filtered out. Thirdly, the coding potential was estimated by the Coding-Potential Assessment Tool (CPAT) [94], Coding Potential Calculator (CPC2) [95], Flexible Extraction of LncRNAs (FEELnc) [96] and the Pfam 31.0 [97] databases. Survived transcripts without coding potential, i.a., CPC (score < 0); FEELnc (coding potential < 0.558); CPAT (cutoff < 0.43) and Pfam (e-value 10^−5^), passed to further analyses. Moreover, transcripts with small noncoding RNAs Rfam [98] annotations were removed from the final predicted lncRNA dataset (novel/unannotated and annotated with lncRNA biotype). We investigated the expression profiles of the predicted lncRNAs within noncoding differential expressed TARs. Similarly to the analysis of DEGs, three binomial tests (Cufflinks, DESeq2, edgeR) with a cut-off adjusted *p*-value < 0.05 were used to identify the differentially expressed lncRNAs (DELs). To uncover the relationship between lncRNAs and mRNAs, *trans*–and *cis*-regulatory elements were investigated. *Trans* lncRNA-mRNA connections were determined based on Pearson’s correlation coefficient (r > 0.9 or r < −0.9) calculation of expression level using R software. We examined the *cis*-acting lncRNAs positioned from 2 kbp distance of the gene using FEElnc and R softwares.

### 4.5. Alternative Splicing Events

To enrich the transcriptomic analysis, we predicted alternative splicing (AS) events by two methods: a super-fast pipeline for alternative splicing analysis (SUPPA v.2) [99] and replicate multivariate analysis of transcript splicing (rMATS v.3.2.5) [100]. Using equal length (90 bp) paired-end reads and Binary Alignment Map (BAM) files, we calculated the percent of splicing inclusion (PSI) for all splicing events. In the SUPPA method, we retrieved uniquely mapped reads from BAM files and remapped paired-end reads to the reference transcriptome using Salmon software [101]. Differential alternative splicing (DAS) events between the LPS-treated and control groups were statistically tested (FDR < 0.05). Additionally, splicing sites with ΔPSI > 0.1 were classified as significant. Alternative events were divided into five main types by rMATS software: alternative 5′ splice site (A5SS), alternative 3′ splice site (A3SS), mutually exclusive exons (MXE), retention intron (RI) and skipping exon (SE). SUPPA classified splicing events with two more types: alternative first (AL) and last exon (AL). Transcripts, with confirmed DAS events in both methods, were placed within a list of genes regulated by splicing. AS events were visualized in the maser package [102] and rmats2sashimiplot.py scripts [103].

### 4.6. Allele-Specific Expression (ASE) Variants

For the identification of single nucleotide variants (SNVs) changing the allele fraction between the LPS-treated and control samples, the multiple rMATS-DVR [104] and R Bioconductor pipeline were used. Firstly, all BAM files were recalibrated by Picard tool (http://broadinstitute.github.io/picard). Then, the variant calling analysis was performed with rMATS-DVR using golden-standard GATK tool [105]. Obtained data regarding the SNV calling were stored in a VCF file. Disturbed and low-quality SNVs were filtered out according to GATK standard parameters: total depth of base coverage < 10; RMSMappingQuality < 40; QualitybyDepth < 2; MappingQualityRankSum < −12.5; and ReadPosRankSum < −8. Subsequently, a removing procedure eliminated SNVs located in the vicinity of the exon–ntron junction (5 bp range), within regions of bidirectional genes, within paralog regions (50 bp up- and downstream sequences) and simple sequence repeat (SSR) regions identified by GMATo tool [106]. Finally, the SNVs with an alternative allele fraction (AAF) > 0 in at least half RNA-seq libraries and significant changes (ΔAAF > 0.1 and FDR < 0.001) between the LPS-treated and control samples were qualified as ASE variants. An allelic imbalance ratio was confirmed by the chi-square goodness-of-fit test evaluating the frequencies of both nucleotides. All the identified ASEs were annotated by SnpEff [107] and a variant effect predictor (VEP) [108]. All the ASE candidates were plotted by Circos [109]. The ASEs were described by allele and reference fractions, chromosomal position, differences in allele fraction, FDR value, gene and single nucleotide polymorphism (SNP) annotations.

### 4.7. RNA Editing Sites Prediction

The filtration procedure (mentioned above in the ASE section) was extended by a few additional steps to find the RNA editing sites occurring in the porcine endometrium. SNVs with observed AAF in at least three samples were investigated in the RNA editing context. Only canonical A-to-I and C-to-U substitutions were separated for further analysis. SNVs with known SNP annotations (*rs ID*) in the Ensembl SNP database (version 91) were also removed. Assuming that 70% editing efficiency is unlikely, SNVs with AAF > 0.7 in any sample were filtered out. Chromosomal coordinates of the survived SNVs were intersected with Sus scrofa PRE-1 SINE regions using bedtools [110]. The final RNA editing A-to-I dataset was compared with the DREP database (one of few available porcine RNA editing databases [111].

### 4.8. Quantitative Reverse Transcription PCR (qRT-PCR) of DEGs

Simultaneously with the sequencing procedure, RNA from the same six endometrial samples was isolated, as different technical replicates, and rewritten to cDNA. The 7300 Real-Time PCR System (Applied Biosystems, Foster City, CA, USA) was used to validate the expression profiles of DEGs. *ACTB* and *PPIA* were selected as the reference genes. Primers for reference and target genes (*BCAS4*, *MGMT*, *TRAF3*, *VEGFA*, *NOTCH1*, *IFI6*) were projected using Primer Express Software 3 (Applied Biosystems). The qRT-PCR reaction conditions and all primers sequences for the reference and target genes were described in Table S9. PCR reaction mixture in a final volume of 20 uL consisted of 12.5 uL of the Sensitive RT HS-PCR Mix SYBR and 0.24 uL Rox (A&A Biotechnology, Poland), cDNA (20 ng), forward and reverse primers 200 nM (*NOTCH1*, *BCAS4*, *IFI6*, *VEGFA*, *TRAF3*) or 500 nM (*MGMT*) of each, and RNase-free water. Comparative cycle threshold method (ΔΔCT) [112] and geometric means of targets and reference gene expression levels were used for qRT-PCR validation. The results of qRT-PCR were statistically checked by the Student’s *t*-test (*p* < 0.05) using Statistica software (Statsoft Inc., Tulsa, OK, USA).

## Figures and Tables

**Table 1:** Overview of the sequencing and mapping results for the six RNA-seq libraries. CTR 1, 2, 3 refers to the biological replicates of the control porcine endometrium; LPS 1, 2, 3 refers to the porcine endometrium after LPS administration; unique reads refer to the reads mapped to only one location of the porcine genome; multi-mapped reads refer to reads aligned to more than one locus on the reference genome; reads mapped with too many loci refer to the reads mapped more than 20 to multiple loci on the reference genome.

SAMPLE	CTR1	CTR2	CTR3	LPS1	LPS2	LPS3
Raw reads	107,429,702	89,680,918	84,472,314	97,318,676	90,921,644	95,504,908
Trimmed reads	99,350,630	82,942,056	78,186,984	89,746,640	83,948,534	87,774,992
Mapped reads	97,654,932	81,309,628	76,784,982	88,226,984	82,560,754	85,830,024
Uniquely mapped reads	89,527,396	60,499,072	58,793,660	80,185,286	70,781,032	73,217,060
Multi-mapped reads	8,066,184	20,785,528	17,964,626	7,990,104	11,741,434	12,567,688
Reads mapped with too many loci	61,352	25,028	26,696	51,594	38,288	45,276

**Table 2:** Differentially expressed lncRNAs identified in the porcine endometrium after 7 days of subclinical LPS administration. MSTRG—cufflinks/stringtie identifiers on gene level; log2FC—log2 fold change.

Gene_ID	Log_2_(FC)	P-Adj	Chr/Contig	Start	End	Strand	ENSEMBL ID
MSTRG.3558	1.42	2.44E-03	12	47,554,682	47,562,366	−	NA
MSTRG.9270	−0.95	1.41E-02	2	11,923,739	11,924,922	+	NA
MSTRG.15739	−1.38	4.80E-03	6	88,799,040	88,800,470	−	NA
MSTRG.19846	1.86	1.64E-06	AEMK02000682.1	1,430,615	1,441,540	+	ENSSSCG00000035563

**Table 3:** RNA editing sites identified in the porcine SINE regions expressed in the endometrium. Star (*) in chromosome: site column indicates RNA editing site confirmed in DREP database. Variant annotations are described by SnpEff software.

Gene Name	Chrom.: Site	Alt. Allele Freq. in CTR	Alt. Allele Freq. in LPS	Variant Annotation
*FAM214A*	1:119024113 *	0.611, 0.474, 0.222	0.571, 0.333, 0.167	intron_variant
*FAM214A*	1:119024125 *	0.471, 0.389, 0.125	0.333, 0.154, 0.214	intron_variant
*SPPL2A*	1:121133488	0.0, 0.071, 0.0	0.273, 0.333, 0.167	3_prime_UTR_variant
*SPPL2A*	1:121133557	0.304, 0.367, 0.333	0.5, 0.419, 0.263	3_prime_UTR_variant
*SPPL2A*	1:121133571	0.36, 0.447, 0.147	0.2, 0.289, 0.261	3_prime_UTR_variant
*ENSSSCG00000004985*	1:169701294	0.143, 0.154, 0.0	0.375, 0.083, 0.0	3_prime_UTR_variant
*DHRS7*	1:189153076 *	0.125, 0.111, 0.25	0.182, 0.2, 0.071	3_prime_UTR_variant
*DHRS7*	1:189153088 *	0.059, 0.125, 0.286	0.1, 0.25, 0.0	3_prime_UTR_variant
*DENND4C*	1:203449165	0.333, 0.1, 0.2	0.0, 0.0, 0.0	downstream_gene_variant
*DENND4C*	1:203449178	0.333, 0.222, 0.2	0.111, 0.0, 0.0	downstream_gene_variant
*HDHD3*	1:254011510	0.25, 0.167, 0.5	0.5, 0.167, 0.667	upstream_gene_variant
*RC3H2*	1:263824986	0.444, 0.4, 0.333	0.444, 0.25, 0.0	upstream_gene_variant
*ENSSSCG00000038757*	1:66466635	0.167, 0.25, 0.4	0.333, 0.5, 0.429	intron_variant
*ENSSSCG00000038757*	1:66466642	0.429, 0.333, 0.2	0.5, 0.571, 0.167	intron_variant
*ENSSSCG00000038757*	1:66466658	0.25, 0.5, 0.0	0.333, 0.25, 0.0	intron_variant
*TRAF3IP2*	1:77485788	0.167, 0.182, 0.571	0.143, 0.0, 0.143	downstream_gene_variant
*ARL14EP*	2:30295149	0.167, 0.167, 0.143	0.444, 0.0, 0.375	3_prime_UTR_variant
*ARL14EP*	2:30295163	0.1, 0.0, 0.167	0.111, 0.2, 0.0	3_prime_UTR_variant
*TMEM161A*	2:58744433	0.3, 0.3, 0.286	0.125, 0.0, 0.0	3_prime_UTR_variant
*GNG3*	2:9050458	0.333, 0.1, 0.143	0.077, 0.0, 0.0	downstream_gene_variant
*RPS15A*	3:26783904	0.0, 0.375, 0.043	0.188, 0.0, 0.143	3_prime_UTR_variant
*ZNF484*	3:41895871 *	0.375, 0.071, 0.429	0.3, 0.0, 0.333	3_prime_UTR_variant
*CRIPT*	3:93916337 *	0.438, 0.4, 0.333	0.4, 0.333, 0.25	splice_region_variant&intron_variant
*ENSSSCG00000021180*	4:36847416 *	0.563, 0.571, 0.333	0.375, 0.435, 0.333	intron_variant
*RDH10*	4:62570908	0.0, 0.571, 0.375	0.577, 0.543, 0.391	3_prime_UTR_variant
*SFT2D2*	4:82714164	0.143, 0.214, 0.333	0.25, 0.222, 0.0	intron_variant
*SFT2D2*	4:82714165	0.5, 0.462, 0.667	0.5, 0.667, 0.0	intron_variant
*SFT2D2*	4:82714213 *	0.545, 0.609, 0.6	0.214, 0.5, 0.308	intron_variant
*SFT2D2*	4:82714216 *	0.667, 0.227, 0.182	0.267, 0.25, 0.308	intron_variant
*SFT2D2*	4:82714221 *	0.538, 0.364, 0.167	0.176, 0.304, 0.2	intron_variant
*SFT2D2*	4:82714222 *	0.154, 0.091, 0.0	0.235, 0.167, 0.067	intron_variant
*ATF6*	4:88593317 *	0.4, 0.667, 0.2	0.364, 0.25, 0.333	3_prime_UTR_variant
*ENSSSCG00000040782*	4:98777743	0.125, 0.182, 0.4	0.0, 0.636, 0.111	downstream_gene_variant
*ENSSSCG00000040782*	4:98777751	0.385, 0.5, 0.667	0.333, 0.375, 0.444	downstream_gene_variant
*KIAA1551*	5:42263049	0.2, 0.0, 0.143	0.167, 0.364, 0.0	downstream_gene_variant
*KIAA1551*	5:42263098	0.333, 0.222, 0.143	0.182, 0.0, 0.0	downstream_gene_variant
*ENSSSCG00000027085*	6:39367588 *	0.286, 0.259, 0.158	0.261, 0.4, 0.222	3_prime_UTR_variant
*CATSPERG*	6:47279285	0.091, 0.25, 0.0	0.429, 0.111, 0.105	intron_variant
*ENSSSCG00000033351*	6:62013919 *	0.25, 0.25, 0.286	0.111, 0.0, 0.071	3_prime_UTR_variant
*PLOD1*	6:72021487 *	0.286, 0.25, 0.25	0.455, 0.375, 0.25	downstream_gene_variant
*PLA2G7*	7:41487728	0.059, 0.2, 0.059	0.286, 0.0, 0.111	3_prime_UTR_variant
*FCF1*	7:97961485	0.2, 0.0, 0.375	0.5, 0.429, 0.333	downstream_gene_variant
*NDUFC1*	8:87702830	0.182, 0.0, 0.176	0.333, 0.125, 0.333	3_prime_UTR_variant
*PPP1R15B*	9:65058149	0.346, 0.393, 0.344	0.373, 0.221, 0.342	3_prime_UTR_variant
*PPP1R15B*	9:65058152 *	0.156, 0.226, 0.121	0.25, 0.107, 0.158	3_prime_UTR_variant
*AAED1*	10:25443226 *	0.217, 0.194, 0.333	0.167, 0.139, 0.077	3_prime_UTR_variant
*AAED1*	10:25443245 *	0.36, 0.385, 0.522	0.458, 0.395, 0.263	3_prime_UTR_variant
*RSAD1*	12:26775820	0.0, 0.111, 0.0	0.333, 0.143, 0.222	downstream_gene_variant
*ENSSSCG00000011234*	13:18421001 *	0.111, 0.2, 0.143	0.167, 0.077, 0.222	intron_variant
*GFM1*	13:98408126	0.25, 0.0, 0.429	0.333, 0.222, 0.25	upstream_gene_variant
*GFM1*	13:98408170	0.0, 0.077, 0.0	0.111, 0.273, 0.143	upstream_gene_variant
*GFM1*	13:98408190	0.375, 0.292, 0.333	0.111, 0.214, 0.143	upstream_gene_variant
*ENSSSCG00000031589*	14:60792171	0.167, 0.333, 0.333	0.182, 0.0, 0.0	downstream_gene_variant
*ZNF277*	18:33313035	0.143, 0.333, 0.429	0.2, 0.286, 0.182	intron_variant
*ZNF277*	18:33313036 *	0.286, 0.0, 0.429	0.0, 0.286, 0.231	intron_variant
*ENSSSCG00000023820*	18:50580239 *	0.571, 0.0, 0.0	0.273, 0.313, 0.179	downstream_gene_variant
*ENSSSCG00000023820*	18:50580258 *	0.571, 0.364, 0.6	0.625, 0.688, 0.318	downstream_gene_variant

## Data Availability

The datasets analyzed during the current study are available in the ENA database under accession number: PRJEB37538 (https://www.ebi.ac.uk/ena).

## Supplementary Materials

Supplementary Materials can be found at https://www.mdpi.com/1422-0067/21/12/4217/s1. Figure S1. RNA-seq quality control evaluation of cDNA libraries according to (a, b) concentration (ng/µL, nM) and (c–h) size [bp]. Electropherograms were generated on the Agilent High Sensitivity D1000 ScreenTape assay. Table S1. Proportion of base pairs mapped to the porcine genome according to the gene structure. Distribution of bases mapped to the porcine reference genome and assigned to intergenic, intronic, UTR, and coding regions. Table S2. Differentially expressed protein-coding genes (DEGs) identified in the porcine endometrium after 7 days of subclinical LPS administration. Table S3. Gene Ontology enrichment classification of DEGs. *p*-value with cut-off < 0.05; BP—biological process; MF—molecular function; CC—cellular components. Table S4. Differentially expressed lncRNAs (DELs) positively correlated with DEGs. Pearson correlation coefficient (r) cut-off > 0.9. Table S5. Intergenic lncRNAs (lincRNAs) connected with known transcriptionally active regions (TARs) in the distance shorter than 2kb. Table S6. Differentially alternative splicing (DAS) events discovered by SUPPA and rMATS tools. Seven and five alternative splicing event types show imbalance in the percent splicing inclusion (PSI) values investigated by SUPPA and rMATS, respectively. Table S7. DAS genes classified to the Gene Ontology terms. Table S8. Differences in alternative allele fraction (ΔAAF) for significant allele specific expression (ASE) sites identified in the porcine endometrium exposed to subclinical dose of LPS. Table S9. Sequences of primers and reaction conditions used in real-time PCR validation for DEGs and reference genes.

## Abbreviations

lncRNAs	long non-coding RNAs
LPS	lipopolysaccharide
RNA-seq	RNA-sequencing
bp	base pair
NGS	next-generation sequencing
CPAT	Coding-Potential Assessment Tool
CPC	Coding Potential Calculator
FEELnc	Flexible Extraction of LncRNAs
Pfam	protein family database
DELs	differentially expressed lncRNAs
GO	Gene Ontology database
TAR	transcriptionally active region
DEGs	differentially expressed genes
SNV	single nucleotide variant
SNP	single nucleotide polymorphism
PGE2	prostaglandin E2
TNF-α	tumor necrosis factor alpha
IL-1β	interleukin 1 beta
IL-6	interleukin 6
IFNs	interferons
PCA	principal component analysis
FPKM	fragments per kilobase of transcript per million mapped reads
FDR	false discovery rate
AS	alternative splicing
rMATS	replicate multivariate analysis of transcript splicing
DAS	differentially alternative splicing
A5SS	alternative 5′splice site
A3SS	alternative 3′splice site
MXE	mutually exclusive exons
RI	retention intron
SE	skipping exon
AF	alternative first
AL	last exon
PSI	percent splicing inclusion
SSR	single sequence repeat
ASE	allele-specific expression
AAF	alternative allele fraction
TLR	Toll-like receptor
I 5	intron 5
dsRNA	double stranded RNA
SINE	short interspersed nuclear element
A-to-I	adenosine to inosine RNA editing
T4	thyroxine
T3	triiodothyronine
MXC-I	major histocompatibility complex I
RhoGTPases	Rho family of GTPase
MAPK	mitogen-activated protein kinas
NF-κB	nuclear factor kappa-light-chain-enhancer of activated B cells
JNK	c-Jun N-terminal kinases
RIG-I signaling pathway	Retinoic acid-Inducible Gene I signaling pathway
heteroref	heterozygous variants in which reference allele was predominant
heteroalt	heterozygous variants in which alternate allele was predominant
true heterozygotes	heterozygous sites with no predominant variant
GATK	The Genome Analysis Toolkit
ΔPSI	difference in PSI values
BP	biological process
MF	molecular function
CC	cellular components
ΔΔCT	comparative cycle threshold method
rs ID	SNP annotation in database
C-to-U	cytidine to uridine RNA editing
VEP	Variant Effect Predictor
VCF	variant calling format
r	Pearson’s correlation coefficient
KEGG	Kyoto Encyclopaedia of Genes and Genomes database
GO	Gene Ontology
ENA	European Nucleotide Archive
