# Supplementary material for: Transcriptome, Spliceosome and Editome Expression Patterns of the Porcine Endometrium in Response to a Single Subclinical Dose of Salmonella Enteritidis Lipopolysaccharide
Source: Int J Mol Sci. 2020 Jun 13;21(12):4217. doi: 10.3390/ijms21124217 (PMC7352703; doi:10.3390/ijms21124217)
Supplement: Supplementary file 1 [file ijms-21-04217-s001.zip › Table9S.docx]

|  |  |  |  |  |  |
| --- | --- | --- | --- | --- | --- |
| **Gene** | **Primers sequences** | **Accession number** | **Primer, nM** | **Reaction Conditions** | **Reference** |
| ***PPIA*** | F: 5'-GCACTGGTGGCAAGTCCAT-3' | U48832 | 300 | 50°C - 2 min°C  Activation: 95°C - 10 min°C  Denaturation: 95°C - 15 s  40  Annealing: 60°C - 1 min | Nitkiewicz et al., 2010 |
|  | R: 5'-AGGACCCGTATGCTTCAGGA-3' |  | 300 |  |  |
| ***ACTB*** | F: 5'-ACATCAAGGAGAAGCTCTGCTACG-3' | U07786 | 500 | Activation: 95°C- 10 min  Denaturation: 95°C - 15 s  Annealing: 61°C - 1 min 40  Elongation: 72°C - 1 min | Spagnuolo-Weaver et al., 1999 |
|  | R: 5'-GAGGGGCGATGATCTTGATCTTCA-3' |  | 500 |  |  |
| ***TRAF3*** | R: 5'- GGACATCTGCTGGTGCATTTG -3' |  | 200 | Activation: 95°C- 10 min  Denaturation: 95°C - 15 s  Annealing: 66°C - 1 min 40  Elongation: 72°C - 1 min |  |
|  | R: 5'- TGGCCTCACGGTATTTACAGG -3' |  | 200 |  |  |
| ***VEGFA*** | F: 5'- TCACCATGCAGATTATGCGGA -3' |  | 200 | Activation: 95°C- 10 min  Denaturation: 95°C - 15 s  Annealing: 66°C - 1 min 40  Elongation: 72°C - 1 min |  |
|  | R: 5'- TGCCTCGCTCTATCTTTCTTTGG -3' |  | 200 |  |  |
| ***MGMT*** | F: 5'- CTTCCAGCAAGAGTCGTTCAC -3' |  | 500 | Activation: 95°C- 10 min  Denaturation: 95°C - 15 s  Annealing: 66°C - 1 min 40  Elongation: 72°C - 1 min |  |
|  | R: 5'- ACCGTTTCTCCAAACTTCACAG -3' |  | 500 |  |  |
| ***NOTCH1*** | F: 5'- GACCACAGGACCCAACTGTG -3' |  | 200 | Activation: 95°C- 10 min  Denaturation: 95°C - 15 s  Annealing: 68°C - 1 min 40  Elongation: 72°C - 1 min |  |
|  | R: 5'- ACTCGTCAATGTTGATGTGGCA -3' |  | 200 |  |  |
| ***BCAS4*** | F: 5'- TTCAGGAACAAGCCGTGGAC -3' |  | 200 | Activation: 95°C- 10 min  Denaturation: 95°C - 15 s  Annealing: 68°C - 1 min 40  Elongation: 72°C - 1 min |  |
|  | R: 5'- AGTCCTCTGTTCTGTACAACGC -3' |  | 200 |  |  |
| ***IFI6*** | F: 5'- CGGTATCGCTCTTCTTGTGCTA -3' |  | 200 | Activation: 95°C- 10 min  Denaturation: 95°C - 15 s  Annealing: 68°C - 1 min 40  Elongation: 72°C - 1 min |  |
|  | R: 5'- AGCGTCTTCTTTTGTCTGTCTCC -3' |  | 200 |  |  |
