# Supplementary figures and images for: Transcriptome, Spliceosome and Editome Expression Patterns of the Porcine Endometrium in Response to a Single Subclinical Dose of Salmonella Enteritidis Lipopolysaccharide
Source: Int J Mol Sci. 2020 Jun 13;21(12):4217. doi: 10.3390/ijms21124217 (PMC7352703; doi:10.3390/ijms21124217)

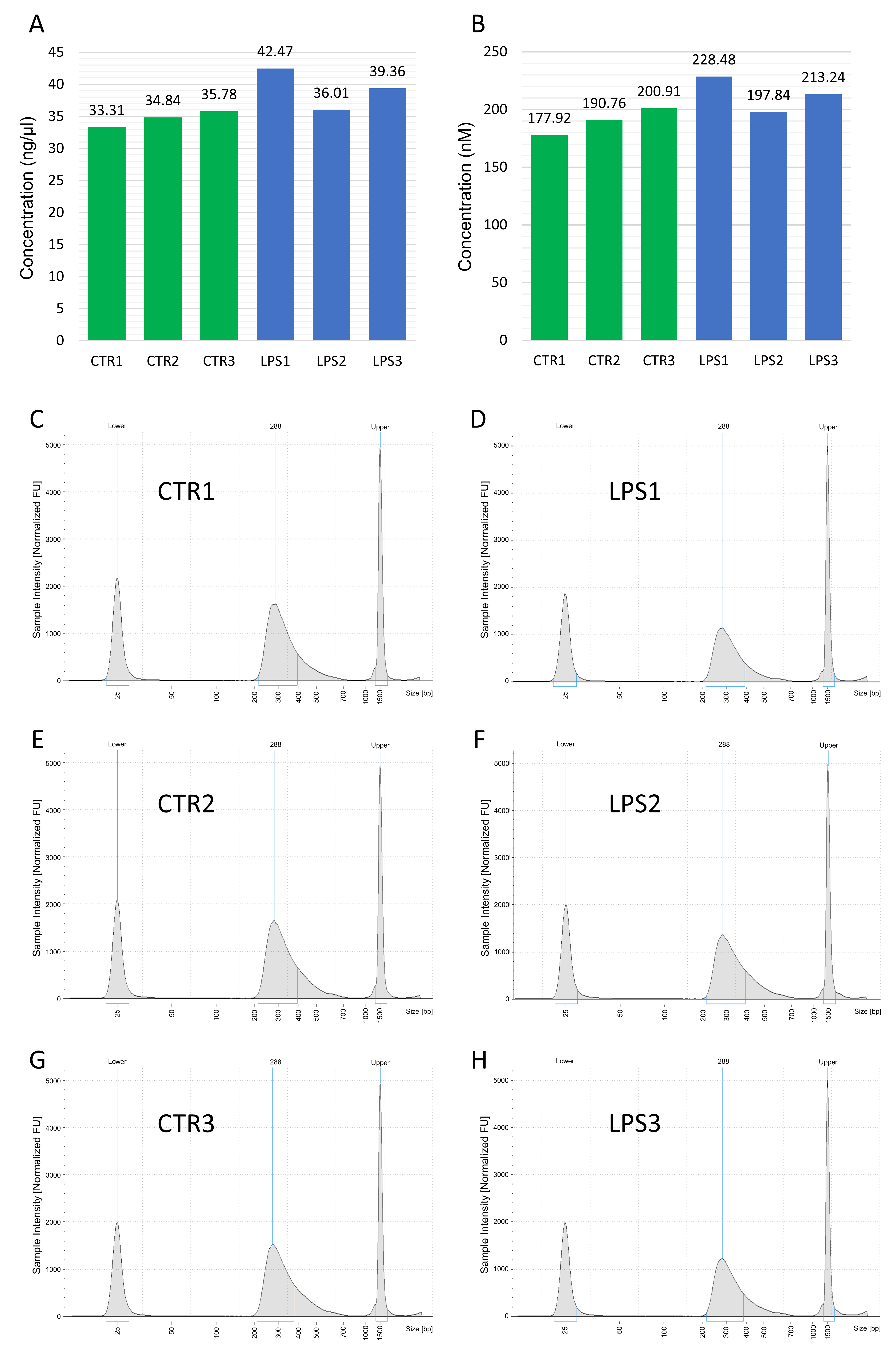

Supplement: Supplementary file 1 [file ijms-21-04217-s001.zip › Figure1S.tif]
